# Nearest neighbor imputation algorithms: a critical evaluation

**DOI:** 10.1186/s12911-016-0318-z

**Published:** 2016-07-25

**Authors:** Lorenzo Beretta, Alessandro Santaniello

**Affiliations:** Referral Center for Systemic Autoimmune Diseases, Fondazione IRCCS Ca’ Granda Ospedale Maggiore Policlinico, Milan, Italy

## Abstract

**Background:**

Nearest neighbor (NN) imputation algorithms are efficient methods to fill in missing data where each missing value on some records is replaced by a value obtained from related cases in the whole set of records. Besides the capability to substitute the missing data with plausible values that are as close as possible to the true value, imputation algorithms should preserve the original data structure and avoid to distort the distribution of the imputed variable. Despite the efficiency of NN algorithms little is known about the effect of these methods on data structure.

**Methods:**

Simulation on synthetic datasets with different patterns and degrees of missingness were conducted to evaluate the performance of NN with one single neighbor (1NN) and with k neighbors without (kNN) or with weighting (wkNN) in the context of different learning frameworks: plain set, reduced set after ReliefF filtering, bagging, random choice of attributes, bagging combined with random choice of attributes (Random-Forest-like method).

**Results:**

Whatever the framework, kNN usually outperformed 1NN in terms of precision of imputation and reduced errors in inferential statistics, 1NN was however the only method capable of preserving the data structure and data were distorted even when small values of k neighbors were considered; distortion was more severe for resampling schemas.

**Conclusions:**

The use of three neighbors in conjunction with ReliefF seems to provide the best trade-off between imputation error and preservation of the data structure. The very same conclusions can be drawn when imputation experiments were conducted on the single proton emission computed tomography (SPECTF) heart dataset after introduction of missing data completely at random.

## Background

The occurrence of missing data is a major concern in machine learning and correlated areas, including medical domains. As the quality of knowledge extracted from data is largely dependent from the quality of data, records with missing values may have a significant impact on descriptive and inferential statistics as well as on predictive analytics.

Missing data can be handled in different ways, but simply ignoring them via deletion methods (e.g. listiwise deltetion) can be an inappropriate choice under many circumstances and besides a general loss of power this may lead to biased estimates of the investigated associations [1, 2]. The replacement of missing values with plausible values derived from the observation of a dataset via imputation procedures is, in most cases, a far better and valuable solution.

Several methods exists for imputing missing data [2–5], among the most popular there are the so-called hot deck imputation methods, that in their deterministic form include the “nearest neighbour” (NN) imputation procedure [6]. In the hot-deck imputation methods, missing values of cases with missing data (recipients) are replaced by values extracted from cases (donors) that are similar to the recipient with respect to observed characteristics. NN imputation approaches are donor-based methods where the imputed value is either a value that was actually measured for another record in a database (1-NN) or the average of measured values from k records (kNN). The most notable characteristics ok NN imputation are: a) imputed values are actually occurring values and not constructed values, b) NN makes use of auxiliary information provided by the x-values, preserving thus the original structure of the data and c) NN is fully non parametric and does not require explicit models to relate y and x, being thus less prone to model misspecification.

Several studies showed that NN may be superior over other hot-deck methods even though results may be dependent from the choice of the metric used to gauge the similarity or the dissimilarity of recipients to donors [7].

Irrelevant or noisy features add random perturbations to the distance measure and hurt performance so that, for instance, points in high dimensional space belonging to the same class (in classification problems) or to the same cluster (in unsupervised clustering applications) have low similarity [8, 9]. The choice of different similarity measures may partially address this issue, but ultimately does not solve the problem [8, 10]. Several methods have been proposed to accommodate the noise and/or to ameliorate the performance of NN algorithms in classification problems, straightforwardly these methods have been applied in imputation problems as well. The use of several k neighbors is a first attempt to control noise and it is widely accepted that small value of k have high influence on the results. kNN proved effective in imputing microarray data with an increased performance, as assessed by the normalized root mean squared error (RMSE), when k is > 1 [11]. In the nearest-variable procedure (kNN-V) and variants (kNN-H and kNN-A) described in [12] k relevant features are selected with respect to the variable with missing values by means of statistical correlation measures; evaluation in real-life and synthetic datasets by means of the RMSE showed a good performance of this method with respect to the selection of neighbors based on intra-subject distance. Other methods that have been proposed to improve the performance of NN to decorrelate error and wade through the noise in classification problems include the use of multiple NN classifiers. In [13] multiple NN classifiers based on random subsets of features are used and the performance of this ensemble was less prone to corruption by irrelevant features as compared to 1-NN or to kNN. Albeit NN is traditionally considered a stable, with low-variance, algorithm that could be not improved by other resampling techniques, such as bagging [14], other experiments indicate that bagging can actually improve the performance of NN provided that the resampling size is adequately below a minimum threshold [15]. Despite these premises, the performance of ensemble methods for NN imputation has not been assessed so far.

A critical and often overlooked point in the evaluation of imputation methods, is the effect the imputed datum has on data structure and on the consequent risk of distorting estimates, standard errors and hypothesis tests despite an apparent good performance on other quality metrics. Imputed data are thus not necessarily more useful or usable and while there are situations where changing the distribution is not of concern, in many cases changing the underlying distribution has a relevant impact on decision making.

In the present paper we assessed the performance of the NN algorithm and modifications in synthetic as well as in real-life datasets, quantifying the effect imputation yields on the data structure and on inferential and predictive statistics.

## Methods

### Frameworks for imputation

NN algorithms are similarity-based methods that rely on distance metrics and results may change in relation to the similarity measure used to evaluate the distance between recipients and donors. In our work, we used the Minkowski norm as metric to evaluate distance:

$$ {\left({\displaystyle {\sum}_{i=1}^n{\left|{x}_i-{y}_i\right|}^p}\right)}^{1/p} $$

The Minkowski norm assumes the form of the Euclidean or L_2_ distance when *p* = 2 or the form of the Manhattan (city-block) distance when *p = 1;* other fractional norms for *p* < 1 have been described [8]. In our experiments we set *p* = 0.5, 1 or 2. In the present paper we only focused on imputation problems with continuous or dichotomous variables, hence there was no need to consider other similarity measures for categorical or ordinal data as described in [16].

Three main NN variants were used for evaluation: a) 1-NN, with one donor selected per recipient, b) kNN with *k* = 5 donors per recipient and, c) weighted wKNN method with *k* = 5 and weighting in relation to the distance of the full set of donors to the recipient as described by Troyanskaya et al [11].

The three NN algorithms were then applied to these frameworks:

- *Plain NN framework*: the full set of data is used according to the hot-deck method and only complete cases with no missing data *C(X)* are considered as donors.
- *Filtered NN framework:* before imputation of the recipient *X*_*i*_, the full set with no missing data *C(X)* is filtered to select a subset of features relevant to the missing variable to be imputed (*X*_*i_miss*_). To this end, *C(X)* is considered as a dataset in the context of a regression problem, where the variable with the missing datum (*X*_*miss*_) is set as the class variable and the other *q* variables (*X*_*1*_*, X*_*2*_*, …, X*_*q*_) as predictors. Since in real-life situations there is usually no clue as to whether any relation exists between predictors and outcome or, if this relation exists, what form it takes, a fully non-parametric selection algorithm is considered an appropriate choice. In the present context, we applied the RReliefF algorithm described in [17]; the set is then filtered to select a subset *C*_*s*_*(X)* ⊂ *C(X)* where (*X*_*1*_*, X*_*2*_*, …, X*_*s*_) ⊂ (*X*_*1*_*, X*_*2*_*, …, X*_*q*_) and *s* < *q*. In the present context we set the number of neighbors for RReliefF equal to 10 and set *s* as 10 %, 20 % or 30 % of *q*. As *C(X)* is invariant to *X*_*i*_, the filtering step is performed only once before the NN imputation step that, on the contrary is performed separately for each *X*_*i*_.
- *Bagged NN framework:* for each recipient *X*_*i*_, from the set of donors with no missing data *C(X)* with size *m,* a random subset of *donors* with size *m*_*R*_ *< < m* is selected with replacement such as *C*_*R*_*(X)* ⊂ *C(X);* the subset *C*_*R*_*(X)* is then used to impute the missing value for the recipient. The procedure is repeated *n* number of times with different random subsets of donors so that several possible values *X*_*iR1,*_*X*_*iR2,*_*…*_*,*_*X*_*iRn*_ are derived from each *C*_*R1*_*(X)*, *C*_*R2*_*(X)*,…, *C*_*Rn*_*(X)* random subsets; the final imputed values for *X*_*i*_ is calculated as the arithmetic mean of the *X*_*iRn*_ imputed values. In our experimental setup *m*_*R*_ is set to 10 % of *m* and *n to* 50 random runs. The random procedure of bagging, provided that *m*_*R*_*/m →* 0 [15], is expected to be helpful in a dataset with several noisy variables that affect the evaluation of the distance from the recipient *X*_*i*_ to the donors. As a result of noise, cases that are dissimilar from *X*_*i*_ with respect to the attributes correlated with the missing variable to be imputed (*X*_*i_miss*_), are factiously selected as “close” to the recipient when they actually lie “far” from it. Bagging the donors with the abovementioned constrains, would help to eliminate such donors and to wade through the noise generated by irrelevant features correcting in part the overly-strong simplicity bias in the NN learner because NN is making incorrect assumptions about the domain and error is reduced by changing it [18].
- *Random NN framework:* for each recipient *X*_*i*_ a random subset of attributes *l* such as that *l* = $\sqrt{q}$ is selected and the corresponding set of donors with no missing data in the *l* attributes *C*_*L*_*(X)* ⊂ *C(X)* is then considered. The procedure is repeated *n* number of times with different random subsets of *attributes* so that several possible values *X*_*iL1,*_*X*_*iL2,*_*…*_*,*_*X*_*iLn*_ are derived from each *C*_*L1*_*(X)*, *C*_*L2*_*(X)*,…, *C*_*Ln*_*(X)* subsets; the final imputed values for *X*_*i*_ is calculated as the arithmetic mean of the *X*_*iLn*_ imputed values. In our experimental setup *n* is set to 50 random runs. Overall, this frameworks differs from c) in that randomization is introduced in the selection of attributes rather in the selection of donors. The repeated random selection of attributes would for each random run favour the removal of irrelevant attributes that may bias the distance metric from recipient to donors and thus lead to unreliable proximities. The procedure is expected to have a high chance of success when in a dataset irrelevant (noisy) attributes outnumber the non-noisy attributes correlated with the missing variable to be imputed (*X*_*i_miss*_) as the odds are in favour of keeping non-noisy variables after the random selection of few attributes. During each random run we do expect to partially control the noise so that it is accommodated to a meaningful extent by the ensemble as the “true” imputation values derived from informative voters outnumber the “random” or “false” imputation values derived from non-informative voters [13].
- *Full Randomized NN framework*: the procedures described in c) and d) are combined together in a random forest-like fashion [19]. This way a double randomization is introduced in the learning algorithm: firstly a random subset of *l* = $\sqrt{q}$ attributes is selected from the set of donors with no missing data *C(X)* so that *C*_*L*_*(X)* ⊂ *C(X)* and then a random subset of donors with size *m*_*R*_ *< < m* is selected with replacement from *C*_*L*_*(X)* to generate a fully randomized subset *C*_*RL*_*(X)* ⊂ *C*_*L*_*(X)* ⊂ *C(X).* The procedure is repeated 50 times and, as in c) and d), the final imputed value is the arithmetic mean of values derived from the 50 random runs.

The five frameworks used to evaluate the NN algorithms were tested against a mean imputation method where the missing value is replaced by the variable mean of complete cases.

### Simulated datasets

To evaluate the performance of the imputation algorithms described above, we generated simulated dataset with a known pattern of missing at random data. In the present context we considered both m*issing completely at random (MCAR)* and *missing at random (MAR)* patterns of randomness [4]. The first refers to the case when the probability of an instance (case) having a missing value for an attribute does not depend on either the known values or the missing data; the latter refers to the case when the probability of an instance having a missing value for an attribute depends on observed values, but not on the value of the missing data itself.

To generate the simulated dataset we firstly created a population of 1*10^6^ individuals with well-known dependencies among attributes and with a linear relationship with a variable *Y* such that:

1 $$ Y=50 + {\beta}_0{X}_0 + {\beta}_1{X}_1 + {\beta}_2{X}_2 + {\beta}_3{X}_3 + \upepsilon $$

where β_0_ = log [2], β_1_ = log [4], β_2_ = log (0.5) and β_3_ = log (0.25) and ε is a random error normally distributed with μ = 40 and ϭ = 4. *X*_*0*_ and *X*_*3*_ are drawn from a multinormal random distribution that encompasses another variable *X*_*4*_ and whose parameters are: *X*_*0*_*,* μ = 200 and ϭ = 40, *X*_*3*_*,* μ = 50 and ϭ = 10, *X*_*4*_*,* μ = 100 and ϭ = 20, *X*_*0*_*by X*_*3*_ ρ = 0.75, *X*_*0*_*by X*_*4*_ ρ = 0.6, *X*_*3*_*by X*_*4*_ ρ = 0.5. *X*_*1*_ is drawn from a multinormal random distribution with one variable *X*_*5*_ and whose parameters are: *X*_*1*_*,* μ = 20 and ϭ = 4, *X*_*5*_*,* μ = 500 and ϭ = 100, *X*_*1*_*by X*_*5*_ ρ = 0.5. *X*_*2*_ is drawn from a non-central chi-square distribution with μ = 20 and 3 degree of freedom.

Two additional variables that are used to generate MAR data were then added to the population. *X*_*6*_ is drawn from a Bernoulli distribution with *p = 0.5* and *X*_*7*_ is drawn from a uniform distribution in the range [30, 60].

Fifty additional variables drawn from different distributions were finally added to the dataset to generate a random noise and whose setting parameters were randomly generated from uniform distributions. These comprise: 15 variables drawn from a non-central chi square distribution with μ = [10, 50] and degree of freedom = [2, 5], 20 normally-distributed variables with μ = [10, 200] and ϭ = μ/5, 3 blocks of 3 variables drawn from a multinormal distribution with μ = [10, 100] and ϭ = [μ/5, μ/2] and 3 blocks of 2 variables drawn from a multinormal distribution with μ = [10, 100] and ϭ = [μ/5, μ/2].

From the population of 1*10^6^ individuals, 400 cases were randomly drawn. Randomness was then introduced for variables *X*_*0*_, *X*_*1*_ and *X*_*2*_, so that each variable had 15 % or 30 % of missing cases. Missingness for *X*_*1*_ and *X*_*2*_ was introduced with a *MCAR* schema removing the desired number of cases in relation to a Bernoulli distribution with *p* = 0.15 or 0.30. Missingness for *X*_*0*_ was introduced according to a *MAR* schema so that it was dependent from *X*_*6*_ (binomial with equal probability) and *X*_*7*_ (uniform in the range [30, 60]). Four dummy categories were created, A: *X*_*6*_ = 0 and *X*_*7*_ < 60th percentile of [30, 60], B: *X*_*6*_ = 1 and *X*_*7*_ < 60th percentile of [30, 60], C: *X*_*6*_ = 0 and *X*_*7*_ ≥ 60th percentile of [30, 60], D: A: *X*_*6*_ = 1 and *X*_*7*_ ≥ 60th percentile of [30, 60]; each category was given a different risk of having missing cases so that Risk (A) = 1, Risk (B) = 1.5 * Risk (A), Risk (C) = 1.5 * Risk (B) and Risk (D) = 1.5 * Risk (C).

The sampling procedure was repeated 500 times for each sample size/percentage of missingness.

### Evaluation of results

After imputation, several estimators from the sampled sets $\widehat{\theta}$ can be calculated and compared with the true parameters *θ* observed in the whole population of 1*10^6^ individuals. From these we can calculate the Bias $\left(\widehat{\theta},\theta \right)$ the variance, Var $\widehat{\theta}$ and the mean squared error, MSE $\left(\widehat{\theta}\right)$ which are defined as follows:

$$ Bias=\left(\overline{X}-\theta (X)\right),\overline{X}=\frac{1}{n}{\displaystyle {\sum}_{i=1}^n{x}_i\kern3.5em Var=\frac{1}{n-1}{\displaystyle {\sum}_{i=1}^n\left({x}_i-\overline{X}\right)\kern3.5em MSE=\frac{1}{n}{\displaystyle {\sum}_{i=1}^n{\left({x}_i-\theta (X)\right)}^2}}} $$

Where X is the measure of interest, more specifically we considered:

- The regression coefficients for the variables with missing values in equation (1) as calculated by the least squared method: β_0_, β_1_ and β_2_.
- The correlation between expected *Ŷ* values calculated inserting the derived regression coefficients and the values of *X*_*0*_, *X*_*1*_, *X*_*2*_ after imputation into equation (1) and the true *Y* values.
- The mean of *X*_*0*_, *X*_*1*_ and *X*_*2*_.
- The standard deviation of *X*_*0*_, *X*_*1*_ and *X*_*2*_.

Additionally, we provided a measure of inaccuracy of imputation defined as the mean of the proportional difference between true and imputed values in the *n* variables with missing values, *n*_*miss*_:

$$ Inaccuracy=\frac{1}{n_{miss}}{\displaystyle {\sum}_{i=1}^{n_{miss}}\frac{\left|\widehat{x}-{X}_i\right|}{X_i}} $$

### Real-life dataset

The performance of the different learning frameworks was finally tested in a real-life dataset. To this end, we considered the single proton emission computed tomography (SPECT)-F heart dataset described by Kurgan et al [20] (available at: https://archive.ics.uci.edu/ml/datasets/SPECTF+Heart). The dataset describes SPECT data in 267 patients with suspected coronary artery disease and comprises a continous outcome and 44 continuous attributes from SPECT images in 22 regions of interest at rest and after stress, respectively termed F1R, F2R,…, F22R and F1S, F2S,…, F22S.

To mirror the evaluation procedure described for synthetic datasets, we firstly determined the regression coefficients of a binary logistic regression equation modelled using a stepwise entry method (entry *p* = 0.01, exit *p* = 0.05). The final model thus obtained was as follow: Y = 44.133 – 0.94 * F5S - 0.123 * F13S - 0.207 * F16S - 0.201 * F20S. A 15 % of random missingness was then introduced in the 4 variables included in the regression equation via the MCAR schema.

The MSE was chosen as the primary performance measure to evaluate the ability of the different learning frameworks to impute missing values. To this end we considered a) the ability to correctly infer the regression coefficients for the variables of interest, b) the inaccuracy in the imputed values, c) the distortion of data as assessed by the standard deviations of the 4 variables. Overall the MCAR/imputation procedure was repeated 500 times; only the wKNN method was used to learn the imputed values and k was set equal to 1, 3 or 10 neighbors.

To summarize the performance of the different learning frameworks and to establish the trade-off between inaccuracy of imputation and distortion of data we proceeded as described hereafter. The mathematical mean between the normalized inaccuracy values (across the different frameworks) and the normalized standard deviation values (across the different frameworks) was calculated for each variable and ranked from the lowest (the best) to the highest (the worst). The average rank of the four variables was then ranked to produce a readily interpretable summary value.

## Results

Our analysis was conducted to individuate the NN procedure that, at the same time, produced the best imputation performance with the minor distortion in the distribution of data. Under this view, all the distance metrics we considered produced the same results and the same conclusions can be drawn for the different *p* values we tested; for sake of brevity hereafter we’ll only show the results obtained with the Euclidean distance.

Table 1 summarizes the effect of the different NN algorithms/learning frameworks on the regression coefficients for the variable of interest *Y*. kNN algorithms had the overall best performance as assessed by the MSE; results are independent from the mechanism of randomness and can be observed both for MAR (β_0_) and MCAR (β_1_ and β_2_) data. Among the different frameworks, the best results were obtained when noisy variable were filtered via RReliefF. The results obtained by resampling methods seem to be independent from the number of neighbors in the kNN algorithm and usually perform slightly worse than less computationally expensive methods.

**Table 1:** Regression Coefficients, average of 500 samples of *n* = 400, k = 5, 15 % or 30 % of missing data

		β_0_						β_1_						β_2_					
	Missing	15 %			30 %			15 %			30 %			15 %			30 %		
Framework	Method	Bias	Var	MSE	Bias	Var	MSE	Bias	Var	MSE	Bias	Var	MSE	Bias	Var	MSE	Bias	Var	MSE
Plain	*1NN*	0.1899	0.0012	0.0373	0.3277	0.0014	0.1088	0.1915	0.0265	0.0631	0.3496	0.0464	0.1685	-0.1065	0.0058	0.0171	-0.2156	0.0084	0.0549
	*kNN*	0.0927	0.0005	0.0091	0.1817	0.0009	0.0339	0.045	0.0181	0.0201	0.0638	0.0425	0.0465	-0.0225	0.0039	0.0044	-0.0589	0.0076	0.011
	*wkNN*	0.0925	0.0005	0.0091	0.1811	0.0009	0.0337	0.0451	0.0181	0.0201	0.0639	0.0425	0.0465	-0.0227	0.0039	0.0044	-0.0594	0.0076	0.0111
RReliefF10	*1NN*	0.158	0.0017	0.0267	0.3025	0.003	0.0944	0.1901	0.0252	0.0613	0.3672	0.0415	0.1763	-0.1003	0.0046	0.0146	-0.2083	0.0082	0.0516
	*kNN*	0.0737	0.001	0.0064	0.1587	0.0025	0.0277	0.0462	0.0176	0.0197	0.0824	0.0384	0.0451	-0.0227	0.0038	0.0043	-0.0602	0.0073	0.0109
	*wkNN*	0.0734	0.001	0.0064	0.1584	0.0025	0.0276	0.0463	0.0175	0.0196	0.0827	0.0384	0.0452	-0.0228	0.0037	0.0042	-0.0602	0.0073	0.0109
RReliefF20	*1NN*	0.1604	0.0012	0.0269	0.298	0.0023	0.0911	0.1803	0.0241	0.0566	0.3594	0.0443	0.1734	-0.0976	0.0047	0.0142	-0.2097	0.0073	0.0513
	*kNN*	0.0715	0.0007	0.0058	0.153	0.0017	0.0251	0.0407	0.0174	0.019	0.0701	0.0399	0.0447	-0.0208	0.0036	0.0041	-0.0607	0.0072	0.0108
	*wkNN*	0.0712	0.0007	0.0057	0.1524	0.0017	0.0249	0.0408	0.0173	0.0189	0.0707	0.0399	0.0448	-0.0209	0.0036	0.0041	-0.0609	0.0071	0.0108
RReliefF30	*1NN*	0.1617	0.0012	0.0274	0.2982	0.0018	0.0908	0.183	0.0221	0.0555	0.3468	0.0438	0.164	-0.0986	0.0049	0.0147	-0.2005	0.0081	0.0483
	*kNN*	0.073	0.0006	0.0059	0.1533	0.0013	0.0248	0.0383	0.0155	0.017	0.0633	0.0387	0.0426	-0.0201	0.0036	0.004	-0.0565	0.0068	0.01
	*wkNN*	0.0727	0.0006	0.0058	0.1527	0.0013	0.0246	0.0384	0.0155	0.017	0.064	0.0388	0.0428	-0.0202	0.0036	0.004	-0.0565	0.0068	0.01
Bagging	*1NN*	0.0812	0.0004	0.007	0.1593	0.0008	0.0262	0.0182	0.0176	0.0179	0.002	0.0394	0.0393	-0.007	0.0038	0.0039	-0.0244	0.0073	0.0079
	*kNN*	0.0869	0.0004	0.008	0.168	0.0008	0.029	0.0095	0.0176	0.0176	-0.015	0.0394	0.0395	-0.0006	0.0039	0.0039	-0.0098	0.0074	0.0075
	*wkNN*	0.0857	0.0004	0.0078	0.1651	0.0008	0.028	0.0096	0.0175	0.0176	-0.0153	0.0392	0.0394	-0.0008	0.0039	0.0039	-0.0104	0.0073	0.0074
Random	*1NN*	0.0874	0.0004	0.0081	0.1589	0.0007	0.0259	0.0121	0.018	0.0181	-0.0114	0.0387	0.0388	-0.0003	0.0041	0.0041	-0.0122	0.0074	0.0076
	*kNN*	0.0872	0.0004	0.008	0.1572	0.0007	0.0254	0.0081	0.0177	0.0177	-0.0212	0.0385	0.0389	0.0014	0.004	0.004	-0.007	0.0074	0.0074
	*wkNN*	0.0872	0.0004	0.008	0.1571	0.0007	0.0253	0.0081	0.0177	0.0177	-0.0212	0.0385	0.0389	0.0014	0.004	0.004	-0.007	0.0074	0.0074
Bagging + Random	*1NN*	0.0939	0.0005	0.0093	0.1686	0.0007	0.0291	0.0128	0.0187	0.0188	-0.0109	0.0388	0.0388	-0.0004	0.0041	0.0041	-0.0101	0.0074	0.0075
	*kNN*	0.0956	0.0005	0.0096	0.1715	0.0007	0.0301	0.0103	0.0185	0.0186	-0.0178	0.0393	0.0395	0.001	0.0041	0.0041	-0.006	0.0075	0.0075
	*wkNN*	0.0953	0.0005	0.0095	0.1708	0.0007	0.0299	0.0102	0.0185	0.0185	-0.0179	0.0392	0.0394	0.0011	0.0041	0.0041	-0.006	0.0075	0.0075
Mean Imputation		0.109	0.0005	0.0124	0.1913	0.0008	0.0373	0.0108	0.0196	0.0197	-0.0147	0.0407	0.0409	0.0015	0.0044	0.0043	-0.0047	0.0078	0.0078

When the correlation between estimated and true values of the dependent value *Y* were considered, the very same conclusions can be drawn (Table 2). The general good performance of kNN algorithms in the context of inferential statistics with the *plain or filtering framework,* is justified by the higher degree of accuracy (e.g. by the lower inaccuracy) these methods have compared to the competitors in imputing the missing value (Table 3). As expected, the inaccuracy of imputation for *X*_*0*_ was lower than the inaccuracy observed for *X*_*1*,_ due to the higher number of dependencies in the simulated datasets; accordingly, *X*_*3*_ that was completely unrelated to other variables and had a non-normal (chi-square) distribution, had the highest degree of inaccuracy in the imputed values.

**Table 2:** Correlation between expected and actual values of the dependent variable Y as calculated from equation [1], average of 500 samples of *n* = 400, k = 5, 15 % or 30 % of missing data

		Estimated vs actual Y					
	Missing	15 %			30 %		
Framework	Method	Bias	Var	MSE	Bias	Var	MSE
Plain	*1NN*	0.16957	0.00072	0.02947	0.30959	0.00114	0.09698
	*kNN*	0.10961	0.00035	0.01236	0.21987	0.00068	0.04902
	*wkNN*	0.10947	0.00035	0.01233	0.21958	0.00068	0.04890
RReliefF10	*1NN*	0.15170	0.00075	0.02377	0.29608	0.00163	0.08928
	*kNN*	0.10036	0.00042	0.01049	0.20963	0.00104	0.04498
	*wkNN*	0.10023	0.00041	0.01046	0.20947	0.00104	0.04492
RReliefF20	*1NN*	0.15246	0.00063	0.02387	0.29331	0.00136	0.08738
	*kNN*	0.09850	0.00035	0.01005	0.20607	0.00087	0.04333
	*wkNN*	0.09839	0.00035	0.01002	0.20580	0.00087	0.04322
RReliefF30	*1NN*	0.15202	0.00068	0.02379	0.29081	0.00122	0.08579
	*kNN*	0.09893	0.00033	0.01012	0.20508	0.00079	0.04285
	*wkNN*	0.09877	0.00033	0.01008	0.20474	0.00079	0.04271
Bagging	*1NN*	0.10287	0.00030	0.01088	0.20756	0.00063	0.04370
	*kNN*	0.10608	0.00030	0.01156	0.21240	0.00061	0.04572
	*wkNN*	0.10544	0.00030	0.01142	0.21078	0.00061	0.04503
Random	*1NN*	0.10629	0.00030	0.01160	0.20738	0.00059	0.04359
	*kNN*	0.10626	0.00030	0.01159	0.20638	0.00058	0.04317
	*wkNN*	0.10622	0.00030	0.01158	0.20631	0.00058	0.04314
Bagging + Random	*1NN*	0.11010	0.00031	0.01243	0.21258	0.00060	0.04579
	*kNN*	0.11101	0.00032	0.01264	0.21422	0.00060	0.04649
	*wkNN*	0.11083	0.00032	0.01260	0.21386	0.00060	0.04633
Mean Imputation		0.11857	0.00035	0.01441	0.22512	0.00063	0.05130

**Table 3:** Inaccuracy in the imputation of missing variables, average of 500 samples of *n* = 400, k = 5, 15 % or 30 % of missing data

		Inaccuracy of imputed values					
	Missing	15 %			30 %		
Framework	Method	X_0_	X_1_	*X*_2_	X_0_	X_1_	*X*_2_
Plain	*1NN*	0.20513	0.22972	0.56589	0.20740	0.23113	0.56644
	*kNN*	0.16135	0.18078	0.45804	0.16467	0.18252	0.46226
	*wkNN*	0.16119	0.18072	0.45814	0.16443	0.18246	0.46245
RReliefF10	*1NN*	0.18585	0.22663	0.56361	0.19424	0.23259	0.56324
	*kNN*	0.14861	0.17971	0.45634	0.15461	0.18305	0.46181
	*wkNN*	0.14846	0.17968	0.45643	0.15447	0.18305	0.46184
RReliefF20	*1NN*	0.18808	0.22416	0.56186	0.19247	0.22891	0.56218
	*kNN*	0.14847	0.17785	0.45450	0.15313	0.18181	0.45896
	*wkNN*	0.14831	0.17781	0.45463	0.15291	0.18179	0.45918
RReliefF30	*1NN*	0.18835	0.22493	0.55807	0.19371	0.22806	0.55949
	*kNN*	0.14918	0.17770	0.45381	0.15377	0.18058	0.45964
	*wkNN*	0.14899	0.17767	0.45394	0.15353	0.18056	0.45974
Bagging	*1NN*	0.15793	0.17242	0.43902	0.16149	0.17539	0.44266
	*kNN*	0.16258	0.17134	0.43148	0.16670	0.17458	0.43561
	*wkNN*	0.16190	0.17119	0.43162	0.16558	0.17431	0.43583
Random	*1NN*	0.16244	0.17164	0.43141	0.16253	0.17400	0.43639
	*kNN*	0.16299	0.17099	0.42932	0.16298	0.17307	0.43380
	*wkNN*	0.16294	0.17097	0.42932	0.16292	0.17306	0.43381
Bagging + Random	*1NN*	0.16635	0.17287	0.43159	0.16636	0.17490	0.43593
	*kNN*	0.16785	0.17238	0.42907	0.16823	0.17436	0.43371
	*wkNN*	0.16765	0.17233	0.42907	0.16799	0.17429	0.43371
Mean Imputation		0.17576	0.17412	0.42819	0.17560	0.17616	0.43270

**Fig. 1:**
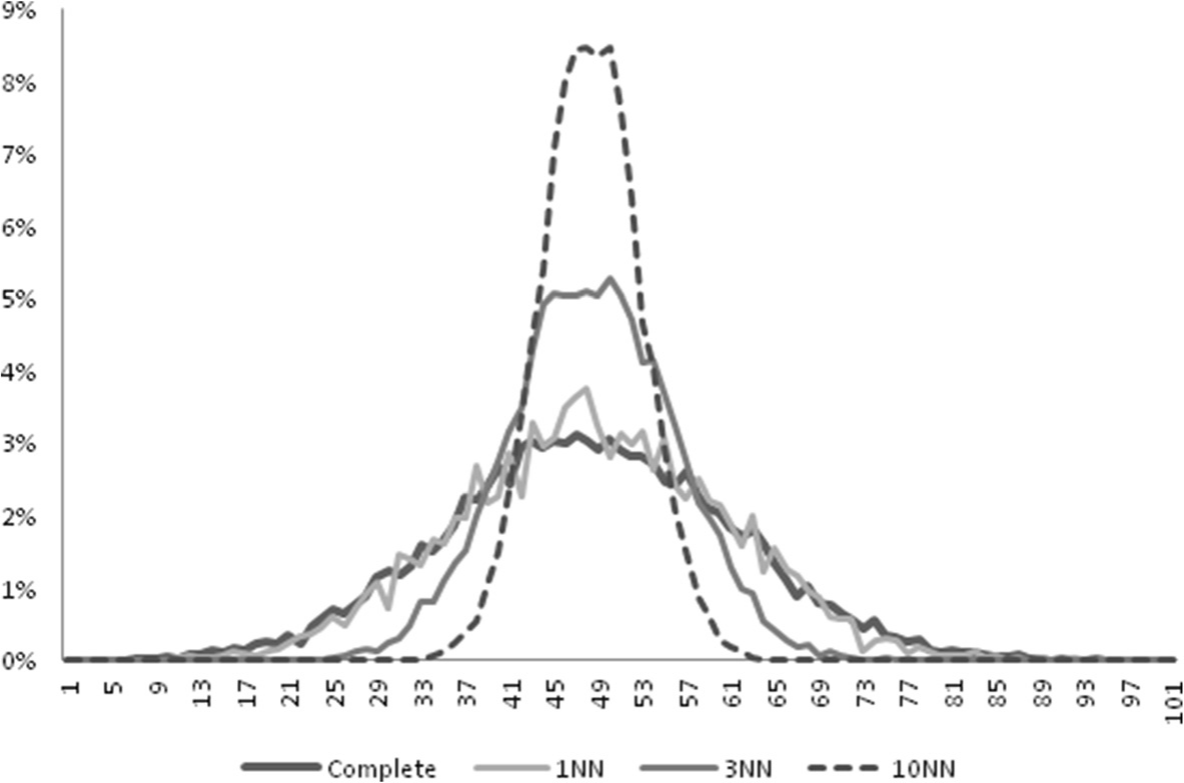
Distribution of data for the variable *X*_*0*_ before removal of missing cases and after imputation with the kNN algorithm, setting k equal to 1, 3 or 10 neighbors

**Fig. 2:**
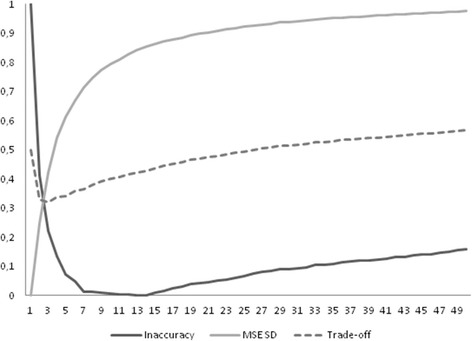
Trade-off between inaccuracy of imputation and MSE of the standard deviation (SD) for the kNN algorithm in relation to the number of k neighbors (x-axis); normalized values are shown; variable of interest: *X*_*0*_

Even if kNN and the other complex methods seem to have a superior performance in inferential statistics as compared to simple 1NN (or 1NN after filtering), these methods caused a not irrelevant distortion of data. The means of the imputed variables were not differentially affected by these methods (results not shown), yet standard deviations were greatly influenced by complexity (Table 4). The detrimental effect on standard deviation is due to a general “flattening” around the mean of the imputed values; it is noteworthy to observe that for resampling schemas the imputed value is indeed very similar to the value obtained by the mean imputation method. The very same effect can be observed with a different degree of magnitude for kNN. Figure 1 plots the distribution of *X*_*0*_ values in absence of missingness and after imputation with k = 1, 3 or 10 neighbors in an additional experiment of 100 imputation runs in samples of size *n* = 400, MCAR = 30 % in the context of the *plain framework* with the kNN algorithm. As it can be clearly observed, only 1NN preserves the original variability in the distribution of data while the distribution of *X*_*0*_ is gradually distorted as the number of k increases. To evaluate the trade-off between inferential statistics and distortion of data we next plotted in Fig. 2 the inaccuracy of imputation vs the MSE of the standard deviation of the mean. As it can be observed, the inaccuracy of imputation decreases as the number of neighbors increases, yet this causes a gradual increase in the MSE of the standard deviation due to an unwanted reduction in the original dispersion of data. The best trade-off between inaccuracy and preservation of data structure, that is the average between normalized inaccuracy and normalized MSE of the standard deviation, is the point where the two curves intersect and corresponds to 3 neighbors. The very same optimal k point could be obtained re-running the experiments in samples with larger sizes (*n* = 1600) or with the filtered NN framework.

**Table 4:** Standard deviation of the mean for the imputed variables, average of 500 samples of *n* = 400, k = 5, 15 % or 30 % of missing data

		X_0_						X_1_						*X*_2_					
	Missing	15 %			30 %			15 %			30 %			15 %			30 %		
Framework	Method	Bias	Var	MSE	Bias	Var	MSE	Bias	Var	MSE	Bias	Var	MSE	Bias	Var	MSE	Bias	Var	MSE
Plain	*1NN*	0.5074	2.3775	2.6302	1.0086	3.5607	4.5708	0.0232	0.0279	0.0284	0.043	0.0423	0.0441	-0.0022	0.1952	0.1948	0.0487	0.3396	0.3413
	*kNN*	2.455	1.9712	7.9943	5.1169	2.2592	28.4371	0.2575	0.0222	0.0884	0.523	0.0238	0.2972	0.5701	0.1469	0.4716	1.2426	0.1643	1.708
	*wkNN*	2.4528	1.9704	7.9826	5.1114	2.2626	28.3844	0.2573	0.0222	0.0883	0.5225	0.0238	0.2967	0.5698	0.1469	0.4713	1.2412	0.1646	1.7049
RReliefF10	*1NN*	0.4393	2.4004	2.5885	0.761	3.3207	3.8931	0.0219	0.0276	0.0281	0.0316	0.0366	0.0375	0.015	0.1709	0.1707	0.0996	0.2514	0.2608
	*kNN*	2.0991	2.0623	6.4642	4.5112	2.533	22.8788	0.2506	0.0228	0.0856	0.5098	0.0249	0.2848	0.5737	0.1449	0.4738	1.2486	0.1575	1.7162
	*wkNN*	2.0946	2.0633	6.4464	4.4987	2.5357	22.7687	0.2504	0.0228	0.0855	0.5091	0.025	0.2841	0.5735	0.1448	0.4734	1.2476	0.1577	1.7138
RReliefF20	*1NN*	0.5633	2.2892	2.6019	0.994	3.2527	4.2343	0.0321	0.0261	0.0271	0.0511	0.0354	0.0379	0.0279	0.1786	0.179	0.1011	0.2649	0.2746
	*kNN*	2.2315	2.0225	6.998	4.72	2.3146	24.5886	0.2541	0.0222	0.0867	0.515	0.0246	0.2898	0.576	0.1437	0.4752	1.2559	0.1563	1.7334
	*wkNN*	2.2275	2.0219	6.9796	4.7088	2.3178	24.4859	0.2539	0.0222	0.0866	0.5143	0.0246	0.2891	0.5757	0.1437	0.4748	1.2545	0.1564	1.7297
RReliefF30	*1NN*	0.6213	2.3558	2.7371	1.0826	3.417	4.5821	0.0321	0.0252	0.0262	0.0606	0.0367	0.0402	0.029	0.1781	0.1786	0.1471	0.2647	0.2858
	*kNN*	2.2891	2.0022	7.2382	4.8352	2.3793	25.7541	0.2557	0.0224	0.0878	0.5193	0.024	0.2936	0.578	0.1455	0.4793	1.2588	0.155	1.7392
	*wkNN*	2.2863	2.0032	7.2263	4.8256	2.3794	25.6606	0.2555	0.0224	0.0876	0.5186	0.024	0.2929	0.5776	0.1454	0.4787	1.2574	0.1551	1.7358
Bagging	*1NN*	2.8371	1.926	9.9715	5.9696	2.0865	37.7185	0.2994	0.0219	0.1115	0.6124	0.0226	0.3976	0.6678	0.1438	0.5895	1.4532	0.1515	2.2629
	*kNN*	3.0206	1.9076	11.0279	6.363	2.0181	42.5012	0.3164	0.0218	0.1219	0.6478	0.0222	0.4418	0.706	0.1432	0.6413	1.5354	0.1473	2.5044
	*wkNN*	3.0141	1.9084	10.9892	6.342	2.0229	42.2391	0.316	0.0218	0.1216	0.6464	0.0222	0.44	0.7051	0.1432	0.6401	1.5322	0.1476	2.4949
Random	*1NN*	2.9856	1.9079	10.8177	6.2409	2.0297	40.9738	0.3132	0.0218	0.1199	0.6386	0.0222	0.4299	0.6984	0.1425	0.63	1.516	0.1472	2.4451
	*kNN*	3.0477	1.9032	11.188	6.3797	2.0142	42.7108	0.3193	0.0218	0.1237	0.652	0.0222	0.4472	0.7123	0.1426	0.6497	1.5472	0.1466	2.5401
	*wkNN*	3.0473	1.9032	11.1854	6.3787	2.0143	42.6979	0.3193	0.0218	0.1237	0.6519	0.0222	0.4472	0.7123	0.1426	0.6496	1.5471	0.1466	2.5397
Bagging + Random	*1NN*	3.0144	1.9068	10.9895	6.3079	2.0163	41.8015	0.3149	0.0218	0.121	0.6423	0.0222	0.4347	0.7015	0.1427	0.6345	1.5238	0.1474	2.469
	*kNN*	3.0742	1.9011	11.3478	6.4416	2.0055	43.4956	0.3205	0.0218	0.1245	0.6548	0.0222	0.4509	0.7141	0.1426	0.6523	1.552	0.1466	2.5549
	*wkNN*	3.0733	1.9012	11.3424	6.4393	2.0056	43.4661	0.3205	0.0218	0.1245	0.6547	0.0222	0.4508	0.7141	0.1426	0.6522	1.5518	0.1466	2.5542
Mean Imputation		3.1024	1.8985	11.5198	6.5012	1.9981	44.2595	0.3224	0.0218	0.1257	0.659	0.0222	0.4564	0.7181	0.1425	0.6578	1.5606	0.1465	2.5817

The capability of the imputation frameworks was finally tested in a real-life dataset with 15 % of MCAR values in 4 variables of interests. As observed in synthetic datasets, the use of several neighbors or of complex learning schemas introduced a non-negligible distortion of data despite a good performance in imputing the correct value or in inferring the regression coefficients (Table 5). Overall, the trade-off between (in)accuracy in imputation and preservation of data was most favourable for few neighbors and when filtering via ReliefF was applied. In this specific setting, the use of 3 neighbors in conjunction with ReliefF 20 % or 30 % yielded the best performance.

**Table 5:** Performance of the different imputation algorithms in the SPECTF dataset with 15 % of cases with missing values (MCAR schema)

		AVG RNK	F5S				F13S				F16S				F20S			
Framework	NN		β	Inacc.	SD	RNK	β	Inacc	SD	RNK	β	Inacc	SD	RNK	β	Inacc	SD	RNK
Plain	*1NN*	11	0.00055	0.10236	0.07645	2	0.00048	0.25751	0.20554	12	0.00188	0.10025	0.25897	14	0.00208	0.08654	0.22260	14
	*3NN*	13	0.00037	0.08654	0.14198	1	0.00026	0.24829	0.55387	15	0.00113	0.09287	0.37754	15	0.00106	0.09324	0.38604	15
	*10NN*	16	0.00044	0.09324	0.29798	6	0.00024	0.25926	0.93204	17	0.00091	0.09282	0.43864	17	0.00085	0.13671	0.67729	17
RReliefF10	*1NN*	12	0.00108	0.13671	0.10879	17	0.00081	0.21663	0.10804	8	0.00245	0.09252	0.21413	9	0.00288	0.11535	0.16061	9
	*3NN*	5	0.00056	0.11535	0.20818	14	0.00038	0.18647	0.25249	5	0.00108	0.08159	0.26951	2	0.00127	0.10922	0.25977	2
	*10NN*	10	0.00038	0.10922	0.36227	16	0.00023	0.18632	0.48904	9	0.00071	0.07924	0.36886	7	0.00076	0.12170	0.48206	7
RReliefF20	*1NN*	4	0.00079	0.12170	0.09823	10	0.00049	0.18938	0.09443	4	0.00177	0.08818	0.19667	4	0.00198	0.10374	0.15313	4
	*3NN*	1	0.00038	0.10374	0.20308	7	0.00023	0.16492	0.24663	1	0.00083	0.07912	0.29916	1	0.00083	0.10194	0.27393	1
	*10NN*	6	0.00030	0.10194	0.34090	13	0.00016	0.16934	0.46077	6	0.00061	0.07827	0.38066	6	0.00054	0.11605	0.49548	6
RReliefF30	*1NN*	3	0.00060	0.11605	0.09507	8	0.00039	0.18664	0.10566	3	0.00153	0.08897	0.21147	5	0.00180	0.09929	0.16041	5
	*3NN*	2	0.00033	0.09929	0.18934	4	0.00019	0.16570	0.26288	2	0.00084	0.08041	0.31290	3	0.00077	0.09905	0.29194	3
	*10NN*	7	0.00027	0.09905	0.32647	9	0.00015	0.17219	0.47827	7	0.00063	0.08011	0.39277	8	0.00052	0.09865	0.51013	8
Bagging	*1NN*	17	0.00052	0.09865	0.36311	12	0.00026	0.26573	1.06013	18	0.00101	0.09273	0.45949	18	0.00084	0.10679	0.79511	18
	*3NN*	20	0.00064	0.10679	0.43314	18	0.00033	0.28017	1.20518	20	0.00117	0.09412	0.48350	20	0.00103	0.12034	0.92381	20
	*10NN*	21	0.00060	0.12034	0.53072	21	0.00042	0.29474	1.32230	21	0.00134	0.09648	0.52562	21	0.00125	0.08574	104.138	21
Random	*1NN*	8	0.00023	0.08574	0.27112	3	0.00013	0.17789	0.61912	10	0.00063	0.07941	0.42192	10	0.00041	0.08773	0.47887	10
	*3NN*	9	0.00025	0.08773	0.31320	5	0.00013	0.18342	0.71124	11	0.00063	0.08053	0.44411	11	0.00040	0.09447	0.55429	11
	*10NN*	14	0.00026	0.09447	0.39598	11	0.00014	0.19570	0.83654	13	0.00064	0.08274	0.46791	12	0.00042	0.10005	0.70133	12
Bagging	*1NN*	15	0.00028	0.10005	0.43214	15	0.00015	0.20818	0.90011	14	0.00068	0.08464	0.47270	13	0.00045	0.10636	0.77000	13
+Random	*3NN*	18	0.00029	0.10636	0.47922	19	0.00016	0.22459	1.02479	16	0.00071	0.08658	0.49146	16	0.00047	0.11750	0.86695	16
	*10NN*	19	0.00034	0.11750	0.52735	20	0.00021	0.25749	1.19241	19	0.00082	0.09074	0.51276	19	0.00056	0.13035	0.96854	19
Mean Imputation		22	0.00060	0.13035	0.59451	22	0.00064	0.30319	1.38048	22	0.00193	0.10097	0.54523	22	0.00244	0.23502	1.11848	22

## Discussion

In the present paper we explored the properties of NN imputation method under different learning frameworks to establish under what circumstances the imputation algorithm yields the best performance in terms of inference and capability to preserve the fundamental nature of data distribution. Overall we showed that: a) NN imputation may have a favourable effect on inferential statistics and that b) the precision of imputation is dependent from the degree of dependencies the variable with missing data has with other variables in the dataset and may be negligible for totally uncorrelated attributes; c) resampling methods do not offer any clear advantage with respect to conventional NN imputation methods; d) ReliefF selection algorithms (RReliefF for continous attributes) may help to wade through the noise and improve the performance of NN algorithms without causing any distortion in the data structure; e) the original data structure is only preserved with 1NN while for any value of k > 1, standard deviations are significantly affected and inflated; f) the use of a small number of k may represent a good compromise between performance and need to preserve the original distribution of data; g) in simulations with medium-sized datasets and a number of noisy variables, the best results in terms of both imputation accuracy and preservation of data distribution can be obtained using kNN with small k in conjunction with ReliefF filtering; h) the very same conclusions as in point g can be reached when a real-life dataset with 15 % of MCAR data is taken into account.

The concept that methods capable of weighting the information provided by different variables may improve the performance of NN has previously been explored in [12] albeit with some substantial differences as compared to our approach. These authors used parametric measures based on the classical Pearson’s correlation to evaluate the distance between the variable to be imputed and the remaining attributes and then borrowed the necessary information from the nearest variables (or in their hybrid approach, by both the nearest variables and the nearest subjects). Compared to the selection by ReliefF family algorithms, this method considers only linear bivariate interactions and may not be the optimal choice when the data structure is completely unknown, underestimating the relevance of attributes in the multidimensional space. In our implementation, variables were selected on the basis of ranking and no weighting on the basis of the attributes’ scores was made. This approach proved effective even selecting a small fraction of variables (e.g. 10 %), yet at the moment we cannot provide any guidance about the optimal number of attributes to select. As previously shown in classification problems [17], this number is context-dependent, however the choice of the most parsimonious model seems reasonable as we do not expect many correlations among variables. Most notably, the selection of the top 20 % or 30 % ranking ReliefF attributes proved effective in obtaining the best performance in a medium-sized real-life dataset with unknown structure or dependencies among variables.

One of the most striking findings of our simulation is that when kNN imputation is chosen, it is advisable to limit the number of k neighbors, because of the risk to severely impair the original variability of data. Again, we cannot provide an optimal number of neighbors to select, however both in the simulations we conducted and in the test in the real-life dataset we subsequently performed, a value of k = 3 seem a reasonable choice. These findings are of paramount importance because contrarily to the common notion derived from the work of Troyanskaya et al [11], a value f k ranging from 10 to 20 may not be appropriate unless data distortion is completely neglected and the accuracy of the imputed data (as measured for instance by the MRSE) is the sole outcome of interest.

In our simulation, we considered a coloured form of noise and we did not just added a “white” Gaussian noise to the variables of interest, including among the irrelevant variables blocks of correlated attributes as well as unrelated attributes normally or diversely (e.g. non central chi-square) distributed and thus our findings are robust against different types of noise. Despite this, the limitations of a simulation setting should be acknowledged as real-life datasets are far more complex and challenging. Even the final test we conducted in the SPECTF heart dataset is not fully exhaustive of what a researcher may encounter in the real-life, as we considered only a MCAR mechanism to create the missing data. The conclusions we draw applies to cases with moderate sizes of missingness, no lower than 15 % and no higher than 30 %; we intentionally limited our evaluations to this range as for small amounts of missing data, under the MAR or MCAR mechanisms, imputation may be useless and for larger amounts caution should always be applied because estimates may become very imprecise [21]. Thus, despite the efficiency of NN imputation under these conditions, it should remembered that imputation should be carefully applied and cannot solve all the problems of incomplete data [22] and that NN imputation can have serious drawbacks as we showed for instance considering the risk of distorting data distribution or the lack of precision in imputing variables with no dependencies in a dataset or, conversely, the possibility to introduce spurious associations considering dependencies where they do not exist.

## Conclusions

The use of ReliefF selection algorithms in conjunction with kNN imputation methods, provided that k are adequately low, gives and adequate trade-off between precision of imputation and capability to preserve the natural structure of data. The use of large number of *k* neighbors is only apparently useful in NN imputation problems as the gain in precision masks a striking distortion in the true distribution of data.

## Abbreviations

MAR, missing at random; MCAR, missing completely at random; MSE, mean squared error; NN, nearest neighbour
